# Ways to increase precision and accuracy of wound area measurement using smart devices: Advanced app Planimator

**DOI:** 10.1371/journal.pone.0192485

**Published:** 2018-03-05

**Authors:** Piotr Foltynski

**Affiliations:** Nalecz Institute of Biocybernetics and Biomedical Engineering, Polish Academy of Sciences, Warsaw, Poland; University of Illinois at Urbana-Champaign, UNITED STATES

## Abstract

**Introduction:**

Wound surface area measurement is important as therapeutic decisions may depend on the change of wound surface area over time. Digital planimetry is a popular technique in wound area measurement. It is accurate and repeatable when calibration is performed with 2 rulers placed at opposite sides of a wound. The aim of the current study was improving accuracy and precision of wound area measurement using capabilities of smart devices.

**Methods:**

The correction of area measurement based on calculated camera tilt angle and the calculation of calibration coefficient of linear dimensions as weighted average were proposed. These and other improvements were applied in the Planimator app for Android, which was then used in the study. Accuracy and precision of the Planimator app were compared to the Visitrak device, the SilhouetteMobile device, the AreaMe software, and to the digital planimetry based on 2-ruler calibration with pictures taken by the smartphone, compact, and D-SLR cameras. Areas of 40 wound shapes of area ranged from 0.14 to 31.72 cm^2^ were measured with each device. Medians of relative errors (REs) were compared in the accuracy tests and standard deviations (SDs) of relative differences (RDs) were compared in the tests of precision.

**Results:**

The median of REs for the Planimator app was not significantly different from the medians of REs for the digital planimetry based on pictures from the compact or D-SLR cameras, but it was significantly lower than the medians of REs for the Visitrak and SilhouetteMobile devices, the AreaMe software and the digital planimetry based on pictures from a smartphone camera. The SD of RDs for the Planimator app was not significantly different from the SDs of RDs for the digital planimetry based on pictures from the compact or D-SLR cameras, but it was significantly lower than the SDs of RDs for the Visitrak and SilhouetteMobile devices, the AreaMe software and the digital planimetry based on pictures from a smartphone camera. The Planimator app installed at a smartphone revealed to be 2-fold more accurate and 1.5-fold more precise than the measurements with using ImageJ software based on pictures taken with the same smartphone.

**Conclusions:**

The Planimator app occurred to have the same accuracy and precision as measurements with digital planimetry with 2-ruler calibration and based on pictures from a compact camera or a D-SLR camera. This app showed better accuracy and precision than the Visitrak and SilhouetteMobile devices, the AreaMe software and the digital planimetry based on pictures from a smartphone camera.

## Introduction

Using a measurement tool in medicine is very important. Ring [1] states that Carl Wunderlich made in second half of 19^th^ century the greatest progress in medicine by his development of the clinical thermometer and the progression of body temperature could be monitored. Simple measurement of body temperature and creating its graphs gave an opportunity to improve the treatment. To measure a progress of treatment one must have a measurement tool, a start point, and an endpoint. In the case of wound closure the lack of wound, e.g. wound with zero surface area, would be the good endpoint and the start point would be the current surface area of wound. Measurement tool must be reliable and giving similar results under consistent conditions. It is obvious that a more reliable method will enable to make a better diagnosis, which is important for the patient. Therefore it is necessary to use and develop more and more reliable measurement methods. As precision is a synonym for reliability, good measurement method should be precise, but not only. Accuracy is also important, and ideal measurement device is both accurate and precise.

Wound surface area measurement has been considered important by the Wound Healing Society, which recommended reevaluating the clinical procedures if the wound does not reduce its surface area by more than 40% in 4 weeks [2]. There are different methods and devices used in wound area measurement [3–6]. The newest methods are based on smart devices [7] or 3D image processing [8, 9].

Wound area measurement with digital planimetry based on wound photographs and pixel counting inside a wound boundary is a cheap and effective method. An accurate and precise result of measurement requires calculation of an averaged calibration coefficient [10]. In a universal graphics software (like free ImageJ software from National Institutes of Health, Maryland, USA), it requires some care and attention. This averaged coefficient is an arithmetic mean from two coefficients from each ruler. They need to put a line segment at the ruler with its ends lying at the centers of ruler ticks at the picture. Next, they need to enter the length of this line segment and note number of pixels per 1 unit of length they use in area measurement. Moreover, users need to do this twice, and then they can calculate the mean number of pixels per 1 unit of length. Calculation of this mean has to be performed outside of the graphics software. After that, the mean coefficient may be entered into a text field and used for area calculation. The entire procedure is error-prone due to many manual operations. It could be simpler in an application designed for wound area measurement.

It was earlier showed that the calibration based on 2 rulers is much more reliable than on one ruler [10]. Two ruler calibration requires an arithmetic mean from coefficients coming from each ruler. This is a very good approach in the case of a wound with regular shape, and when the rulers are at equal distances from edges of the wound. In the case of an irregular wound or unequal ruler-edge distances, a better measurement result would be achieved with a weighted average. A more detailed explanation will be delivered in the next section. It was also stated [10] that “when a camera is not positioned at right angle to the wound plane the reproduction of wound area at the picture is not the same as when this angle is 90°. The reproduced area is proportional to the cosine of this angle. It would be easy to compensate the influence of this angle if we knew this angle, but we don’t.” This angle may be calculated after introducing additional information about the onboard photographic camera in smart devices such as smartphone or tablet. The method will be presented in the next section.

The aim of the current study is to present some ways for increasing accuracy and precision of wound area measurement with digital planimetry when smart devices are used for the measurement.

## Materials and methods

### Calibration coefficient calculated as weighted average

**Fig 1:**
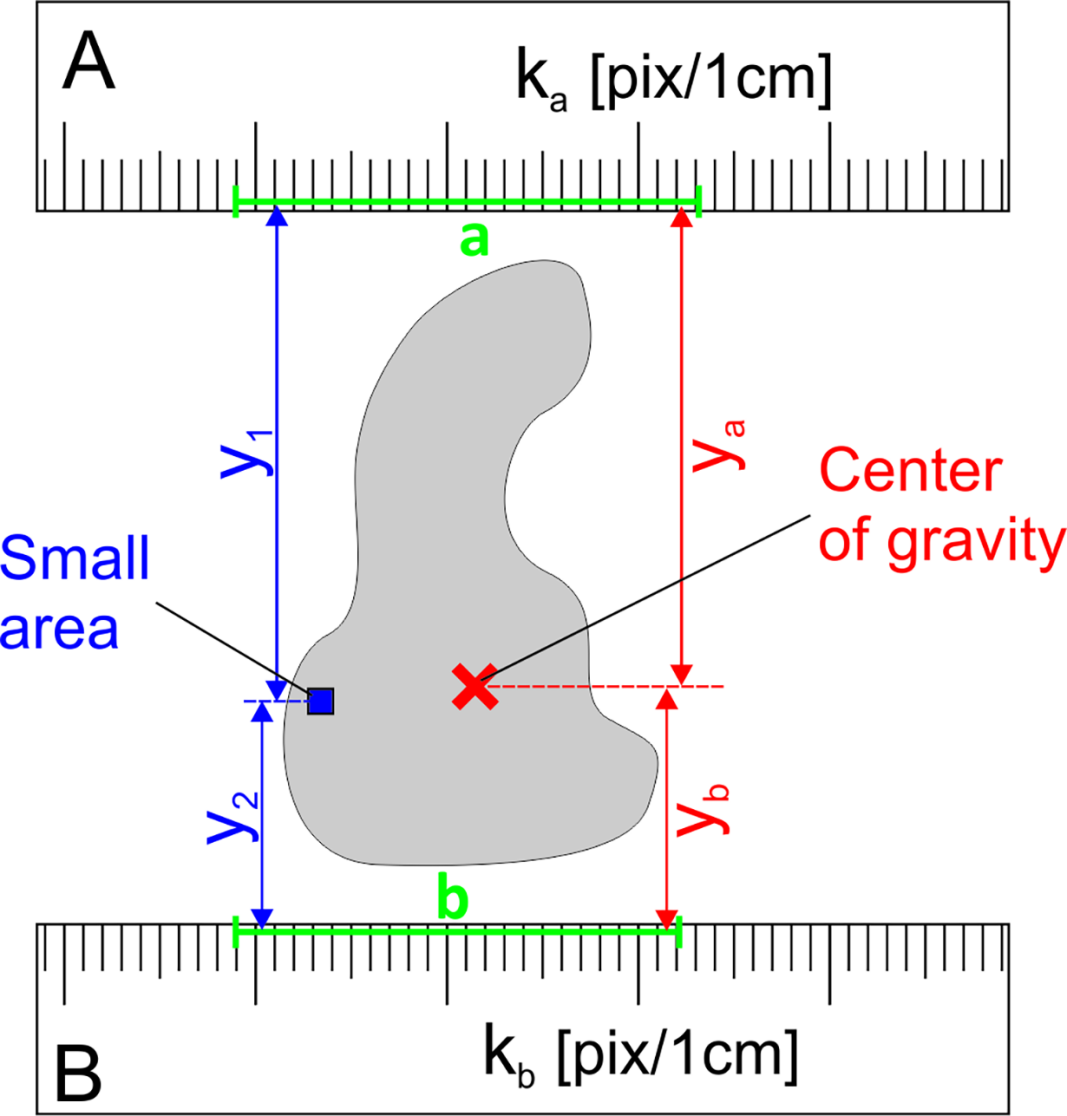
A wound shape with a marked small area and the center of gravity of this shape. The small area is at a distance *y*_*1*_ from the line segment *a*, and at a distance *y*_*2*_ from the line segment b. The center of gravity is at a distance *y*_*a*_ from the line segment *a*, and at a distance *y*_*b*_ from the line segment b. These line segments are used for calculation the calibration coefficients *k*_*a*_ and *k*_*b*_ as number of pixels per 1 cm at ruler A and B, respectively.

During wound area measurement with digital planimetry based on 2-ruler calibration of linear dimensions at the picture, the coefficient k which is the number of pixels per 1 cm, is calculated as arithmetic mean from 2 coefficients from each ruler [10]. Let us consider a small area of the figure which is at the distance *y*_*1*_ from the calibrating line segment *a*, and at the distance *y*_*2*_ from the line segment *b* (Fig 1). The averaged coefficient *k*_*0*_ for this small area should be calculated as weighted average:

$$k_{0}=\frac{{k_{a}y}_{2}+{k_{b}y}_{1}}{y_{1}+y_{b}},$$ (1)

where *k*_*a*_ and *k*_*b*_ are the coefficients calculated from the line segments *a* and *b*, respectively.

**Fig 2:**
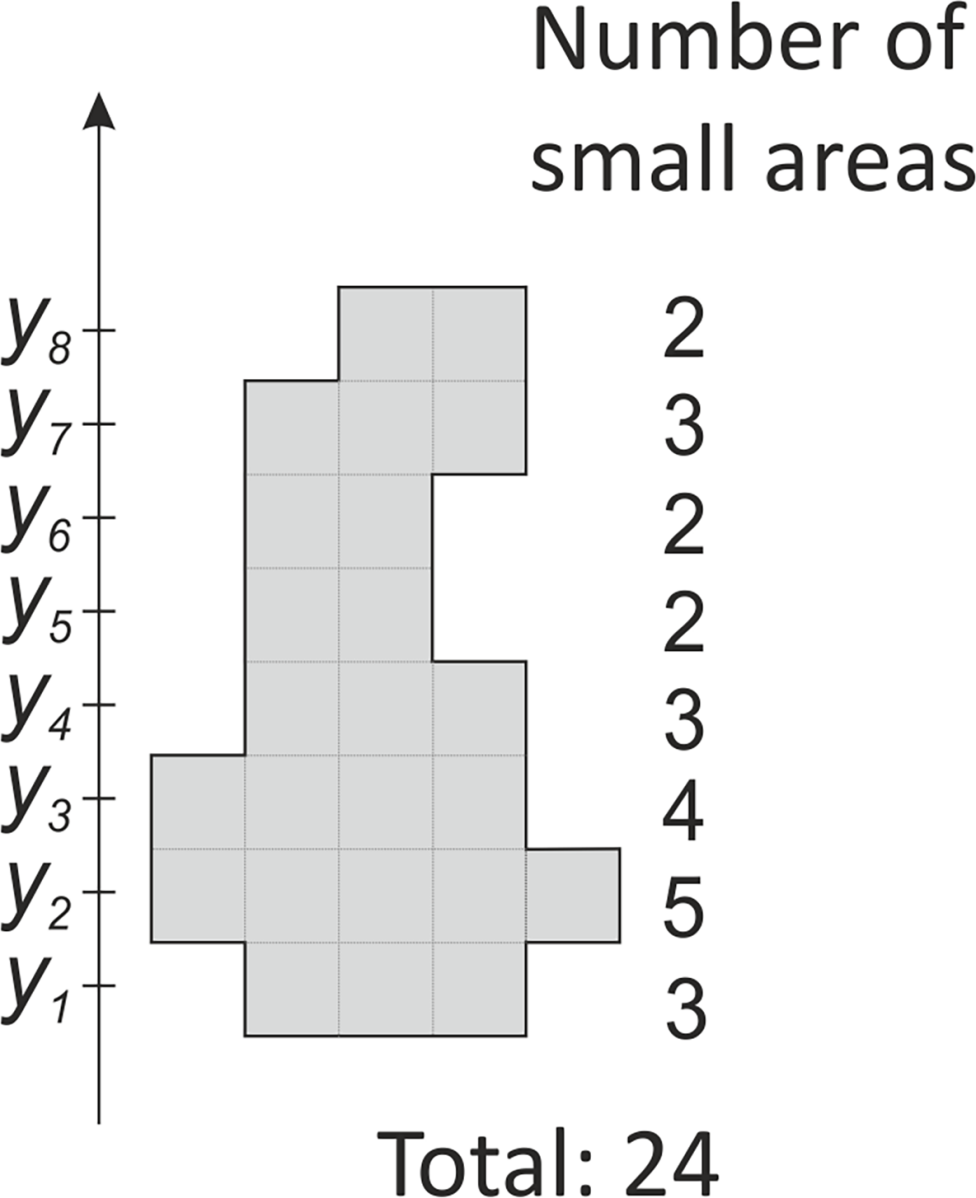
A sample figure (in grey), which was divided into small areas. At each coordinate *y*_*1*_ through *y*_*8*_ there is a variable number of small areas. The total number of the small areas is 24.

When an averaged coefficient is to be calculated for the entire figure, it must be divided into many small areas, as for instance in Fig 2, and the averaged coordinate *y*_*0*_ should be calculated as a weighted average:

$$y_{0}=y_{1}\frac{3}{24}+y_{2}\frac{5}{24}{+y}_{3}\frac{4}{24}+\cdots +y_{8}\frac{2}{24}.$$ (2)

In general, for *k* number of *y* coordinates, it may be written:

$$y_{0}=\sum_{i=1}^{k}y_{i}\frac{n_{i}}{N}=\frac{1}{N}\sum_{i=1}^{k}y_{i}n_{i},$$ (3)

where *N* is the total number of small areas in the figure, and *n*_*i*_ is the number of small areas in the row *i* at the coordinate *y*_*i*_. The right part of Eq (3) is equal to the coordinate of center of mass [11], which in the parallel gravity field, is the same as the center of gravity. The center of gravity of a plane figure is also called the centroid or geometric center. It can be analytically calculated based on the coordinates of its vertices [12]. In the case of wound area measurement, user needs to make a wound outline around the wound at its picture. In real, this outline is a polygon with its vertices lying at wound edges. When the coordinates of the center of gravity are known, the averaged calibration coefficient *k* for the wound outline (Fig 1) may be calculated as:

$$k=\frac{{k_{a}y}_{b}+{k_{b}y}_{a}}{y_{a}+y_{b}},$$ (4)

where *y*_*a*_ and *y*_*b*_ are the distances from the center of gravity to the line segments *a* and *b*, respectively. In the special case when *k*_*a*_ = *k*_*b*_, the *k* is not influenced by the position of the center of gravity. When *y*_*a*_ = *y*_*b*_, the *k* is equal to the arithmetic mean of *k*_*a*_ and *k*_*b*_.

### Calculation of tilt angle

**Fig 3:**
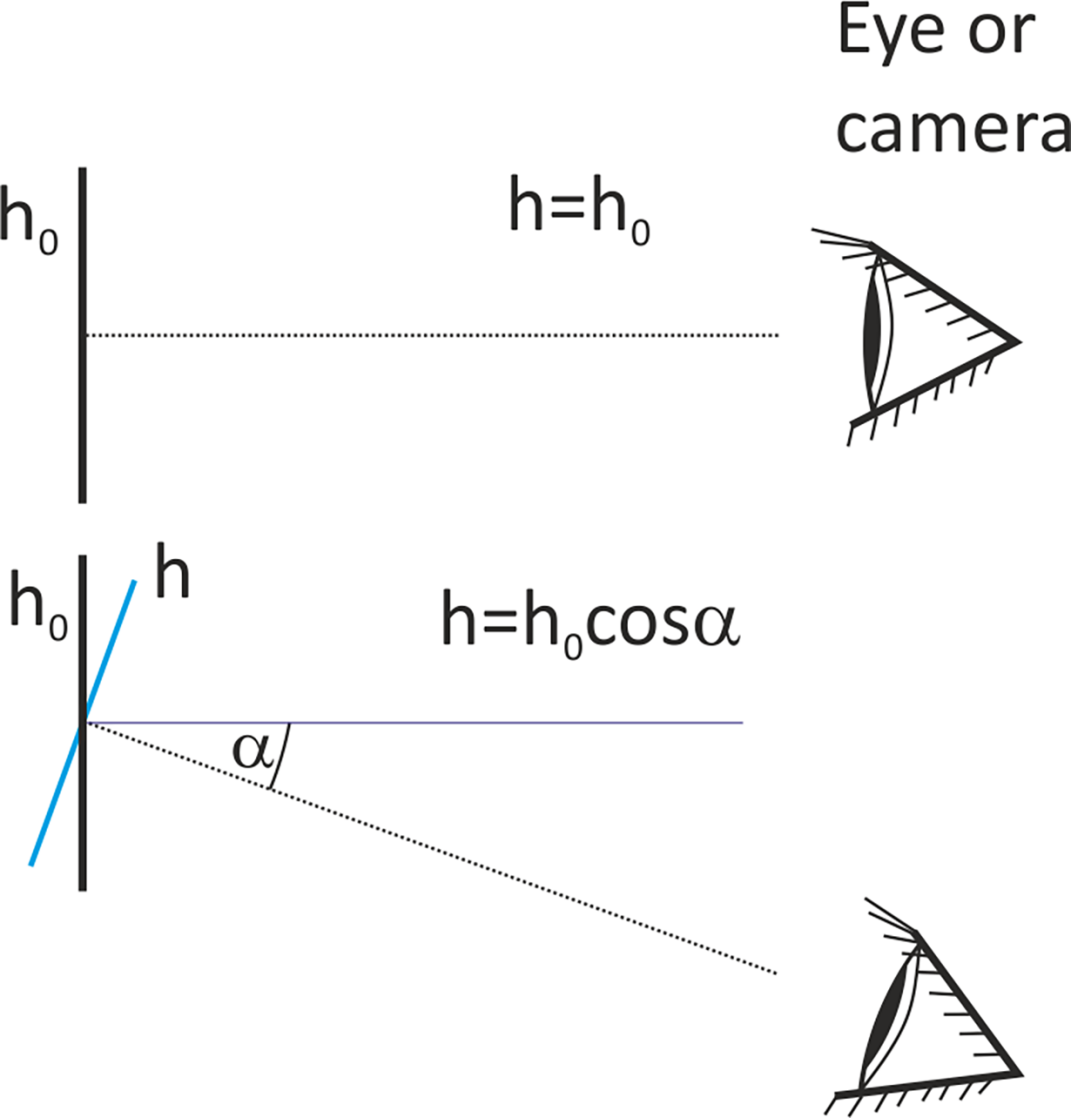
**Height *h***_***0***_ **of an observed object is correctly reproduced at the picture when it is observed at the right angle (upper view).** In the case when there is a deviation from the right angle by the angle *α*, the reproduced height *h* of the object is smaller than *h*_*0*_ (lower view).

The height *h* of an object at the picture in a unit of length is a quotient of the number of pixels along the object *N* and the coefficient of linear dimensions *k*. This coefficient is also called a calibration coefficient, and is expressed in pixels per unit of length. Therefore *h = N/k*, and is expressed the in units of length from this coefficient. This calculated height *h* is equal to the actual height *h*_*0*_ of the object only when the object was photographed without camera tilt i.e. at right angle (Fig 3). When this angle is not 90°, the actual height *h*_*0*_ of the observed object may be calculated as:

$$h_{0}=\frac{h}{cos\alpha },$$ (5)

where *α* is the tilt angle defined as deviation of the observation axis from the normal to *h*_*0*_. After inserting the counted number of pixels *N* and the coefficient of linear dimensions *k*, one can receive:

$$h_{0}=\frac{N}{k}\frac{1}{cos\alpha }.$$ (6)

**Fig 4:**
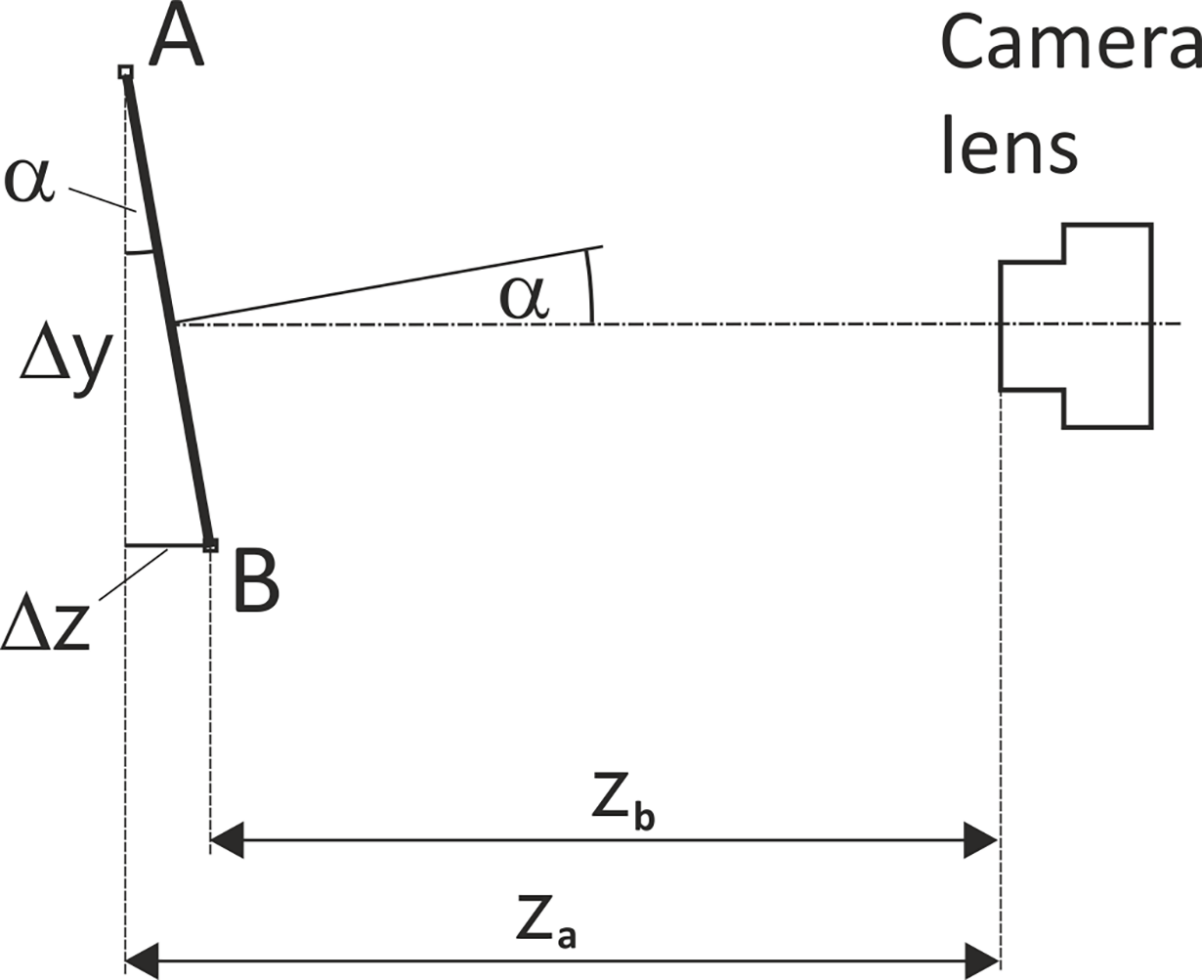
A line segment *AB* observed by a camera. Distance *Δy* is the height of this line segment seen by a camera; *z*_*a*_ and *z*_*b*_ are the distances of the vertices A and B from the camera, respectively, and α is the camera tilt angle.

The Eq (5) can be useful in estimation the actual height *h*_*0*_ of an object at a picture, but the tilt angle *α* should be known. Fig 4 shows a line segment *AB* which is not perpendicular to the principle axis of camera lens, and it can be written that:

$$tan\alpha =\frac{\Delta z}{\Delta y},$$ (7)

where *Δz* is the difference in distance of points *A* and *B* from the camera, and *Δy* is the height of line segment *AB* seen by the camera.

**Fig 5:**
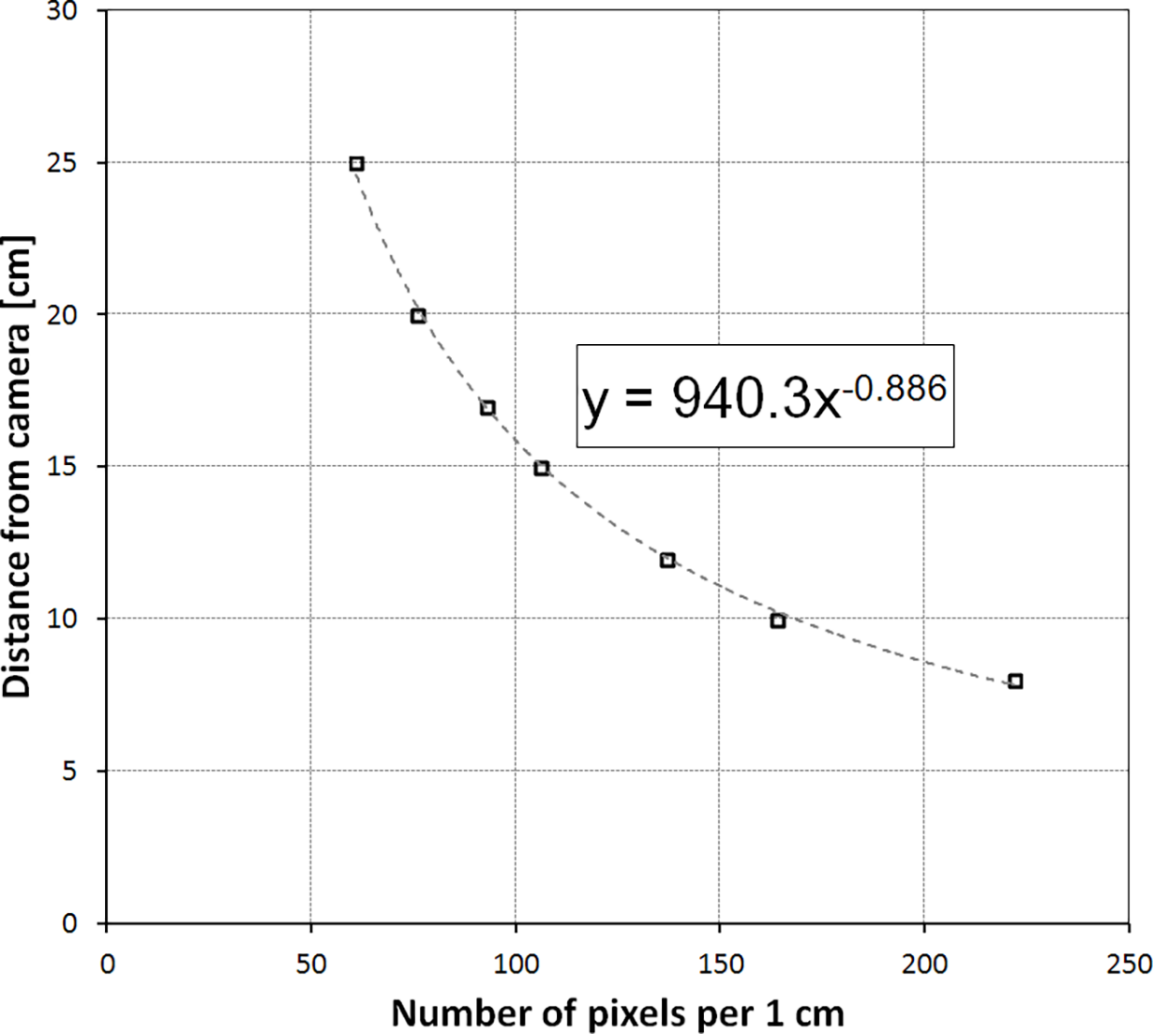
The relationship between the distance from camera and the calibration coefficient *k* for Samsung Galaxy S4 smartphone.

The distance between a certain point and a camera may be easily calculated after finding a mathematical relationship between distance and calibration coefficient which is distance dependent. To get the necessary data, the ruler must be photographed at many different distances, and the calibration coefficient *k* must be calculated from each picture. Then using the curve fitting function the best formulae was found for the relationship of the distance and the calibration coefficient. Power function Y = aX^b^ occurred to fit the best in this case. Fig 5 shows the camera distance equation for the Samsung Galaxy S4 smartphone with parameters *a* and *b* of the power function equal to 940.3 and -0.886, respectively.

Now, the Eq (6) may be written as:

$$h_{0}=\frac{N}{k}\frac{1}{cos\alpha }=\frac{N}{k}\frac{1}{cos(arctan\frac{\Delta z}{\Delta y})}=\frac{N}{k}\frac{1}{cos(arctan\frac{z_{A}-z_{B}}{\Delta y})}=\frac{N}{k}\frac{1}{cos(arctan\frac{\left|f(k_{1})-f(k_{2})\right|}{\frac{N}{k}})},$$ (8)

where *f* is the calibration function of distance from camera; *k*_*1*_ and *k*_*2*_ are the calibration coefficients for distances *z*_*A*_ and *z*_*B*_, respectively; *k* is given by Eq (4).

### Planimator app

In the Planimator app for Android an automatic procedure for calculating the calibration coefficient *k* based on the center of gravity and the correction of area measurement result based on the calculated tilt angle of camera were introduced. Some other advantages of this app are described below. The procedure of finding a mean calibration coefficient from 2 rulers in the ImageJ software is time consuming therefore in the Planimator app it has been simplified. The user needs only to point out a beginning and an end of a line segment at each of two rulers used for calibration of linear dimensions. This takes a few seconds instead of a few minutes as in the case of manual procedure performed in the ImageJ software. The Planimator app automatically recognizes ruler ticks along the line segment and calculates the number of pixels per 1 cm. It also compares the distances between ruler ticks and seeks for the longest sequence of ruler ticks equally distant from the adjacent ticks. This enables to skip artifacts at the ruler which could cause a calibration error.

The area *A* (expressed in square centimeters) in the Planimator app is calculated as

$$A=\frac{N}{k^{2}},$$ (9)

where *N* is the number of pixels within boundary traced around a wound, and *k* is the averaged number of pixels per 1 cm given by the Eq (4). When the tilt angle α can be calculated, the final corrected area *A’* is calculated in cm^2^ as

$$A^{\prime }=\frac{A}{cos\alpha }.$$ (10)

The distance between 2 adjacent ticks at calibration ruler is a parameter in the Planimator app settings. Its default value is 1.0 for a standard ruler with 10 ticks per 1 cm, but if for instance the ruler has got ticks every 0.5 cm, this parameter must be set to 5. This parameter can be also used when paper rulers used for calibration are not accurate. For example, if they show 10.0 cm for 9.9 cm of true distance, this parameter must be set to 0.99, instead of 1.0. Inch rulers also can be used, but the area measurement result will be displayed in cm^2^.

Once the measurement is completed, the Planimator app displays information about percentage change of area for the current wound, taking the first area measurement for this wound as a reference value. This is performed for each unique wound label. All saved area measurement results are stored in a text file with time stamps. The data from this file can be imported to any statistical software or spreadsheet program. When saving numeric data a picture with traced wound outline is also saved and is labeled with the value of measured area and the current date.

### Accuracy and precision of area measurement with the Planimator app

Accuracy and precision of area measurement by the Planimator app were tested in the same way as in previous study [10]. The wound artifacts as grey shapes printed on a white paper were used. Each shape was created in CorelDraw (Corel Corp., Ottawa, Canada) and reflects a real wound or ulcer from a patient with diabetic foot syndrome. Forty wound shapes (range 0.14–31.72 cm^2^) were measured with the Planimator app installed on a Samsung Galaxy S4 smartphone. The results of the Planimator app measurements were compared to the results from the previous study [10] obtained by the Visitrak device, the SilhouetteMobile device, the AreaMe software, and to the digital planimetry based on 2-ruler calibration. Relative differences (RDs) and relative errors (REs) in area measurement were compared. Standard deviation (SD) of relative differences (RDs) is a measure of measurement precision (repeatability). The lower is the SD of RDs the higher is the precision (repeatability). The accuracy of measurement may be assessed by analyzing REs. REs of an accurate measurement method are distributed close to zero, and when the accuracy decreases, the REs move away from zero. The lower is the median of REs the more accurate is the measurement method.

The RD was calculated as a ratio of the difference between the measured and actual areas to the actual area. The RE was calculated as a ratio of the absolute difference between the measured and actual areas to the actual area. The actual value was measured with a reference method based on pixel counting in the images of resolution 600 dpi x 600 dpi from optical scanner. Measurements with the digital planimetry were based on the photographs from the compact, smartphone, and digital single-lens reflex (D-SLR) cameras; ImageJ software was used for area calculations of the printed wound shapes, which were photographed together with 2 paper rulers placed below and above of each wound shape. In these comparisons the same smartphone Samsung Galaxy S4 was used to exclude possible influence of differences between the smartphone cameras in different smartphone models.

### Test of measurement correction based on calculated angle of camera tilt

The efficacy of the correction was assessed after the area measurement of one selected wound shape #3 [13]. This wound shape was measured 12 times with the correction switched on and off. The camera tilt angle was set to 0°, 5°, 10°, 15°, and 20°, and measured with a digital angle measurer. The smartphone Samsung Galaxy S5 attached to a tripod was used.

### Statistical analysis

The Kruskal–Wallis test and the median test were used for comparison of medians of REs in the current study. SDs of RDs were compared and tested using tests for homogeneity of variances: F-test, Bartlett’s, Cochran’s, Hartley’s, and Levene’s tests. Normality of data was tested using Kolmogorov-Smirnov, Lilliefors, and Shapiro-Wilk tests for normality. In tests of the tilt angle correction the mean and the reference value were compared using the *t*-test for testing the sample mean for a difference from the true value. The tests were repeated for each tilt angle with the correction switched on and off. In all tests *P*-value lower than 0.05 was considered statistically significant.

## Results

### Accuracy test

**Fig 6:**
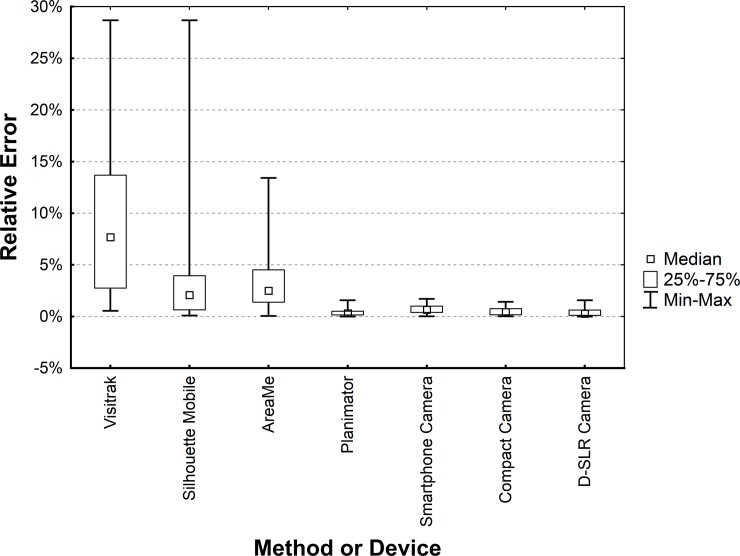
Box-plot of relative errors for the Planimator app and other devices used in the comparison. Smartphone, compact and D-SLR cameras were used with 2-ruler calibration and the area was calculated using ImageJ software.

The median of REs for the Planimator app (Table 1) occurred to be significantly lower than for: (a) the Visitrak device (*P* < 0.0001 for the median and Kruskal-Wallis tests), (b) the SilhouetteMobile device (P < 0.0001 for the median and Kruskal-Wallis tests), (c) the AreaMe software (P < 0.0001 for the median and Kruskal-Wallis tests), and (d) the smartphone camera (P < 0.002 for the median and Kruskal-Wallis tests). The median of REs for the Planimator was not significantly different from medians of REs for the compact camera (*P* > 0.17) and the D-SLR camera (*P* > 0.66). Fig 6 shows distributions of REs for all methods used in the comparison of accuracy.

**Table 1:** The medians and ranges of relative errors for the Planimator app and other methods or devices used in the comparison. Data necessary for the REs calculations are given in the S1 Table.

Method or device	Median of REs^a^	Range of REs^a^
Visitrak	7.69%	0.5%–28.7%
SilhouetteMobile	2.09%	0.09%–28.7%
AreaMe	2.50%	0.05%–13.4%
Smartphone camera	0.66%	0.02%–1.7%
Compact camera	0.42%	0.02%–1.4%
D-SLR camera	0.28%	0.005%–1.6%
Planimator^b^	0.32%	0.0008%–1.6%

^a^ *From N = 40 values for each method or device*.

^b^ *The app was installed at Samsung Galaxy S4, as this smartphone was used in the previous study [10], from which data were taken for comparison.*

### Precision test

SDs of RDs were calculated for all measurement methods and the results are gathered in Table 2. Analysis of homogeneity of variances used for the comparison of SDs revealed significantly lower SD of RDs for the Planimator app than for the Visitrak device (*P* < 0.0001), the SilhouetteMobile device (*P* < 0.0001), the AreaMe software (*P* < 0.0001), and the Smartphone camera (*P* = 0.001). The SD of RDs for the Planimator app was not significantly different from the SDs for the digital planimetry based on pictures from the compact camera (*P* = 0.070) and for the digital planimetry based on pictures from the D-SLR camera (*P* = 0.071).

**Table 2:** Standard deviations (SDs) and ranges of relative differences (RDs) for the Planimator app and other methods or devices used in the comparison. Data necessary for the REs calculations are given in the S1 Table.

Method or device	SDs of RDs^a^	Range of RDs^a^
Visitrak	8.92%	-28.7%–0.7%
SilhouetteMobile	5.83%	-28.7%–4.2%
AreaMe	3.54%	-13.4%–0.1%
Smartphone camera	0.75%	-1.7%–1.2%
Compact camera	0.59%	-1.4%–1.3%
D-SLR camera	0.58%	-1.6%–1.2%
Planimator^b^	0.52%	-1.6%–0.5%

^a^ From N = 40 values for each method or device.

^b^ The app was installed at Samsung Galaxy S4.

**Fig 7:**
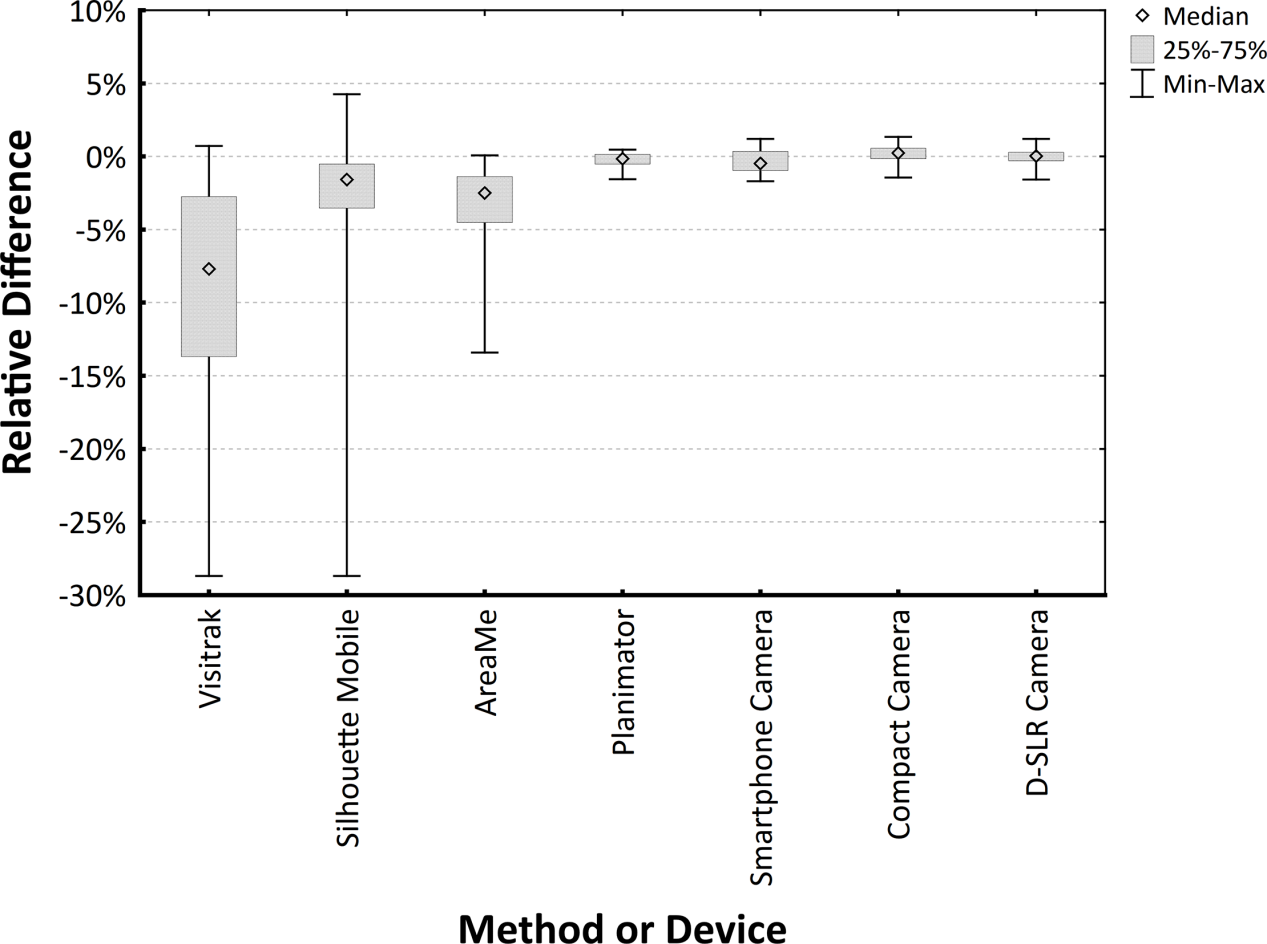
Box-plot of relative differences for the Planimator app and other devices used in the comparison. Smartphone, Compact and D-SLR cameras were used for taking the pictures of wound shapes and the area was calculated using ImageJ software based on 2-ruler calibration.

Fig 7 shows distributions of the RDs for all methods of area measurement used in the comparison of precision.

### Efficacy of correction based on calculated tilt angle

**Fig 8:**
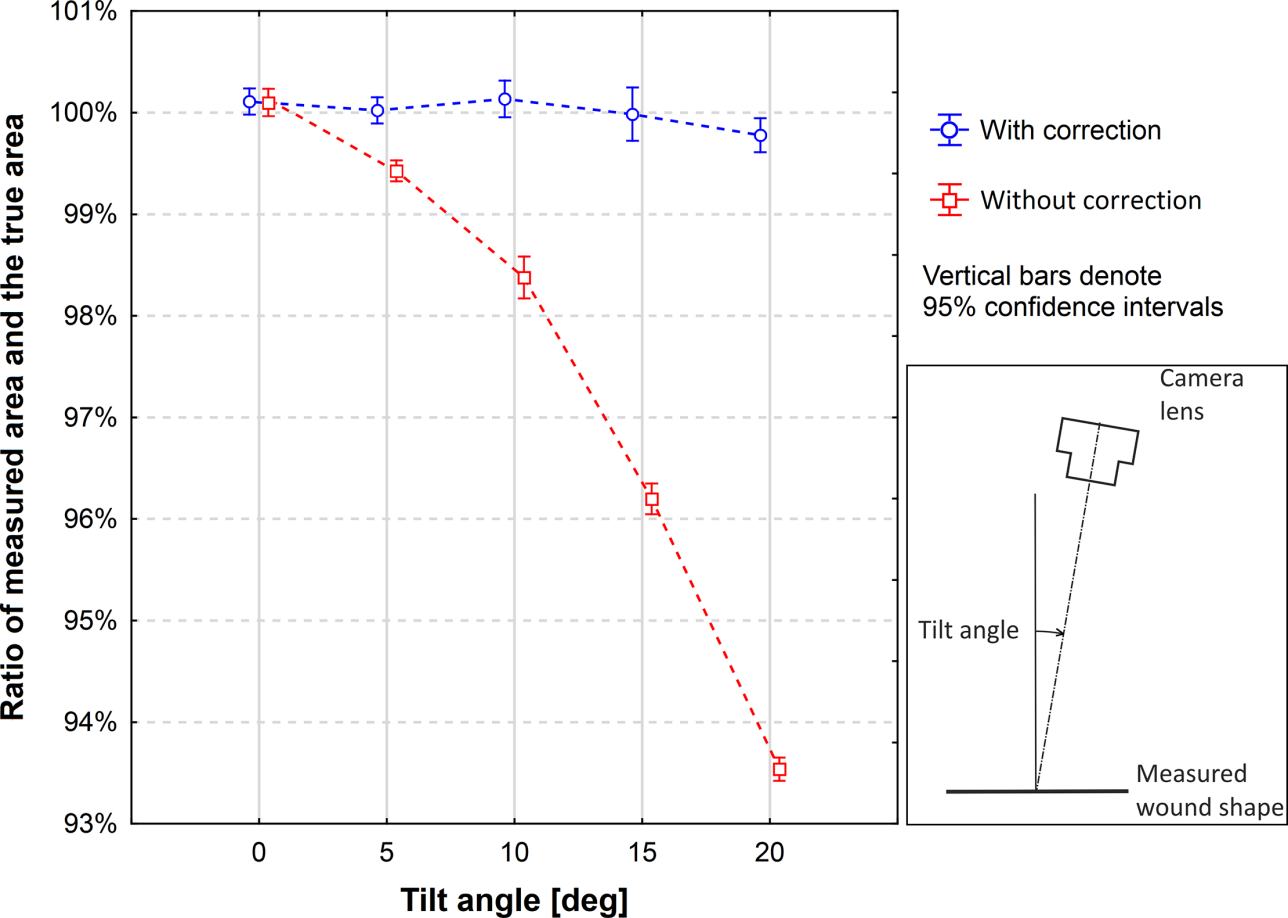
Ratios of the measured area and the true area of a wound shape. The areas of wound shapes were measured with the Planimator app when the measurement result was corrected by the calculated tilt angle and when it wasn’t. The true area of the measured wound shape was 24.027 cm^2^.

The means of measured area with the correction switched on and off were compared to the true value for each tilt angle. For the measurements with the correction switched on there were no significant differences for the angles 0°, 5°, 10°, and 15°, but there was a significant difference for the angle of 20° (Table 3). For the measurements with the correction switched off the differences were nonsignificant for the angle 0°, but they were significant for the other angles (0°, 5°, 10°, 15°, and 20°). Fig 8 shows the ratios of the measured mean value and the true value for 5 angles when the correction was switched on and off. The relative differences of means in respect to the true value and the *P*-values are shown in Table 3.

**Table 3:** RDs of mean of measured area and the true value for 5 tilt angles of camera for the correction switched on or off. *P*-values are given from the *t*-test for comparison the sample mean to the true value. Means were calculated from 12 measurements at each angle. Data necessary for the RDs calculations are given in the S2 Table.

Tilt angle [deg]	Relative difference of mean and the true value (±SD)	
	Correction On	Correction Off
0	0.11(± 0.20)% (*P* = 0.08)	0.09(±0.23)% (*P* = 0.18)
5	0.02(± 0.20)% (*P* = 0.71)	-0.57*(±0.16)% (*P* < 0.0001)
10	0.14(± 0.28)% (*P* = 0.12)	-1.62*(±0.32)% (*P* < 0. 0001)
15	-0.01(± 0.41)% (*P* = 0.91)	-3.80*(±0.24)% (*P* < 0. 0001)
20	-0.22*(± 0.26)% (*P* = 0.014)	-6.46*(±0.18)% (*P* < 0. 0001)

*Denotes significant difference of mean from the true value at P = 0.05.

## Discussion

There were many attempts for more accurate and precise wound area measurement using digital planimetry. In some apps one two-dimensional (2D) marker is used for area calibration [14, 15]. In one patent application [16] the use of 2 or more 2D markers was proposed for area calibration, but an app was not presented. In the case of more than one marker an average area from all markers at the picture was proposed to be used for area calibration. The use of multiple 2D markers may give good accuracy in the case of evenly distributed wounds and when markers are at even distances from wound edges. Moreover, flat 2D markers must lie on the same plane as the measured wound. This is easy to assure on large and flat areas of skin, but it may be problematic on a curved area, where the use of narrow and flexible rulers may bring better measurement accuracy. The use of dedicated calibration markers to an app is of course convenient, because they can be automatically detected in the picture, but the user needs to have a continuous supply of such markers to perform measurements. Thus, the use of standard paper rulers for calibration as in the Planimator app may be a more versatile solution than the use of dedicated markers. Some researchers may try to use the Adobe Photoshop software for wound area measurement with a convenient tool called “magnetic lasso”, which enables fast pixels counting in scanned image of wound outline [17].

The medians of REs for the Visitrak device, the SilhouetteMobile device, the AreaMe software, and the Smartphone Camera were significantly higher than the median of REs for the Planimator app (Table 1), therefore the measurement with the Planimator app is more accurate than with any of these listed before methods. The median of REs for the Planimator app was not significantly different than the medians of REs for the digital planimetry with 2-ruler calibration based on pictures from the Compact and D-SLR cameras, therefore the Planimator app have comparable accuracy as digital planimetry with 2- ruler calibration based on pictures taken with the D-SRL or Compact cameras.

The SD of RDs for the Planimator app was significantly lower than the SDs of RDs for the Visitrak device, the SilhouetteMobile device, the AreaMe software, and the Smartphone camera (Table 2), therefore the Planimator app is more precise than any of these 4 methods used for comparison. As the SD of RDs for the Planimator app was not significantly different than the SDs of RDs for the measurement with the ImageJ software based on pictures taken with the D-SRL or Compact cameras, therefore the Planimator app have comparable precision as digital planimetry with 2-ruler calibration based on pictures taken with the D-SRL or Compact cameras.

The median of REs for the Planimator app was 0.32%, and for the measurement using ImageJ software based on pictures from the Smartphone Camera the median was 0.66%, therefore there is a 2-fold increase in accuracy using the Planimator app. The SD of RDs for the Planimator app was 0.52%, and for the measurement with using ImageJ software based on pictures taken with the same smartphone the SD was 0.75%, therefore the increase in precision of the measurement is about 1.5-fold.

The results of the test of tilt angle correction showed that the means of measured area for the measurements with switched on correction were not significantly different from the reference value for the angles from 0° to 15°, which means that the correction is highly effective up to the angle of 15°. For a larger angle of 20° the correction gives a significant difference, producing a small error of about 0.22%. The measurement with switched off correction produced systematic errors, which increased with the increased tilt angle. The error was equal to about 3.8% for the angle of 15° (Table 3). The value of this error is close to the theoretical value, because *cos*(15°) = 0.9659, and such a tilt of the camera should decrease the height of figure at the picture to about 96.6% of the true figure height and the measured area of figure should be decreased by about 3.4%.

It must be noted that the correction of measured area based on calculated tilt angle and calculation of the weighted coefficient of linear dimensions improve the measurement result only in the case when a picture is taken with a tilt. In the case when the tilt is 0°, they would not be necessary, but the number of cases when a picture is taken at the right angle is very small. The camera is held in hand, and there are not any reference objects to set the camera at the right angle during taking a wound picture. Moreover, patients may have difficulty keeping the body part with a wound in stable position. In consequence, even when users do pay attention to the correct camera position, taking a picture with the tilt of 0° is only a matter of chance. In the majority of cases users are focused on other aspects of taking a picture like for instance focusing or framing. Therefore, when the correction of area measurement is performed automatically the measurement may be more accurate and precise than when performed without the correction, but only with a recommendation that the picture should be taken at the right angle. Some authors pointed out that incorrect angle may cause large measurement error [18,19] up to more than 30% [20], but the way of its correction was not presented.

The Planimator app requires the manual tracing of wound outline and some users may treat it as its weak point. Unfortunately, even commercial devices require such a manual tracing, as the user may trace a wound boundary in the best way when a wound has their boundaries not clearly defined. Automatic algorithms are not efficient in the case of such wounds. Moreover, in the case of the Visitrak device the user must do the manual tracing twice. The first time with a marker on a transparent foil placed on the wound, and the second time with the enclosed electronic pen when the foil is at the device’s sensitive surface.

The advantages and disadvantages of the Visitrak and SilhouetteMobile devices, the AreaMe software based method, and the digital planimetry based on pictures from a Compact, D-SLR and smartphone cameras were discussed earlier [6,7,10], but not all weak points were showed. Those methods of wound area measurement which require the wound tracing on the double-layer foil (like the Visitrak device and the AreaMe software based method) are exposed to a certain issue. The foil placed over an open wound is prone to fogging [19], as it stops the moisture. The fogged foil is less transparent, the user encounters problems with accurate wound margins identification, and the tracing is less accurate. This directly influence the accuracy of wound area measurement.

When disposable paper rulers are used for area measurement with the Planimator app, some deviations from the true area value may occur when the ticks at these rulers are at wrong distances than they supposed to be. This can be noticed when the paper ruler is compared to a very accurate ruler on the caliper, for instance. When the first ticks at both rulers are aligned (e.g. at 0 cm), the ticks at a large distance (e.g. 10 cm) are no more aligned. Such paper rulers cause a systemic bias by under- or overestimation of the measured area. Only verified paper rulers may be used in accurate area measurements.

It was shown in the current study that the proposed correction of area measurement based on the calculated tilt angle is effective. The Planimator app was successfully used in the measurements of surface area of wounds in rats when the induced diabetes was treated with insulin or metformin [21]. Therefore, the Planimator app with the automatic correction of tilt angle influence and the calculation of calibration coefficient based on the center of gravity may be a valuable tool in wound area measurements, which are important in prediction of healing outcomes [22]. Interested readers may watch the video with English subtitles on www.youtube.com showing the wound area measurement with the Planimator app [23].

## Supporting information

S1 Table Results of wound shapes measurements with different methods or devices. (PDF)

S2 Table Results of area measurements of one wound shape with Planimator app for different tilt angles with correction switched on or off. (PDF)
