# Supplementary material for: Ways to increase precision and accuracy of wound area measurement using smart devices: Advanced app Planimator
Source: PLoS One. 2018 Mar 5;13(3):e0192485. doi: 10.1371/journal.pone.0192485 (PMC5837081; doi:10.1371/journal.pone.0192485)
Supplement: S1 Table — (PDF) [file pone.0192485.s001.pdf]

S1 Table. Results of wound shapes measurements with different methods or devices.

ID of wound shape	Reference (true) area of the wound shape [cm^2]	Visitrak [cm^2]	Silhouette Mobile [cm^2]	AreaMe [cm^2]	D-SLR Camera [cm^2]	Compact Camera [cm^2]	Smartphone Camera [cm^2]	Planimator [cm^2]
1	1.2152	1.2	1.2	1.17	1.212	1.211	1.221	1.220
2	0.4105	0.3	0.4	0.37	0.409	0.410	0.410	0.405
3	24.0272	24.2	23.9	23.76	24.249	24.205	23.950	24.077
4	19.2750	19.1	19.3	19.01	19.400	19.356	19.506	19.318
5	1.1087	1.0	1.1	1.06	1.122	1.115	1.098	1.114
6	4.3130	4.2	4.3	4.24	4.353	4.366	4.264	4.314
7	5.7868	5.7	5.7	5.66	5.805	5.816	5.749	5.790
8	4.5932	4.4	4.5	4.48	4.586	4.575	4.539	4.574
9	0.9152	0.8	0.9	0.88	0.916	0.925	0.907	0.919
10	4.3735	4.0	4.4	4.26	4.374	4.432	4.330	4.391
11	3.7463	3.5	3.7	3.72	3.779	3.693	3.721	3.753
12	0.1402	0.1	0.1	0.12	0.138	0.140	0.138	0.140
13	0.2603	0.2	0.2	0.23	0.257	0.259	0.257	0.261
14	0.5422	0.4	0.5	0.51	0.535	0.538	0.538	0.534
15	10.6508	10.3	10.6	10.66	10.722	10.756	10.604	10.592
16	4.7445	4.4	4.7	4.63	4.755	4.757	4.720	4.732
17	1.8776	1.6	1.8	1.84	1.871	1.881	1.862	1.875
18	0.6715	0.6	0.7	0.67	0.668	0.666	0.663	0.661
19	0.8302	0.6	0.8	0.79	0.831	0.835	0.825	0.833
20	2.5021	2.3	2.4	2.39	2.505	2.522	2.479	2.490
21	1.5986	1.4	1.6	1.51	1.602	1.603	1.605	1.600
22	1.0288	0.8	1.0	1.01	1.030	1.029	1.028	1.027
23	5.8485	5.6	5.7	5.72	5.848	5.852	5.790	5.808
24	0.6742	0.6	0.7	0.63	0.670	0.677	0.674	0.673
25	1.9419	1.8	1.9	1.86	1.938	1.934	1.909	1.931
26	1.7756	1.6	1.7	1.70	1.776	1.769	1.777	1.773
27	3.4168	2.9	3.3	3.29	3.417	3.447	3.410	3.400
35	0.4199	0.3	0.4	0.36	0.420	0.420	0.424	0.417
39	4.9513	4.7	4.9	4.84	4.958	4.973	4.970	4.936
50	0.7440	0.6	0.7	0.66	0.741	0.745	0.747	0.744
52	1.6674	1.5	1.6	1.62	1.666	1.670	1.678	1.665
68	1.3416	1.2	1.3	1.33	1.342	1.348	1.356	1.338
70	2.7182	2.7	2.7	2.68	2.707	2.714	2.691	2.704
73	12.3673	12.3	12.1	12.10	12.397	12.357	12.255	12.217
75	1.4416	1.4	1.4	1.40	1.437	1.449	1.440	1.447
80	29.5987	28.9	29.5	29.38	29.635	29.581	29.350	29.386
83	3.2192	3.1	3.2	3.14	3.216	3.228	3.237	3.220
96	15.9624	15.6	16.0	15.77	16.042	16.051	16.028	15.950
101	24.2392	23.9	24.0	24.13	24.390	24.291	24.322	24.148
108	31.7176	30.7	31.6	31.36	31.862	32.030	31.712	31.756
