# Supplementary material for: Ways to increase precision and accuracy of wound area measurement using smart devices: Advanced app Planimator
Source: PLoS One. 2018 Mar 5;13(3):e0192485. doi: 10.1371/journal.pone.0192485 (PMC5837081; doi:10.1371/journal.pone.0192485)
Supplement: S2 Table — (PDF) [file pone.0192485.s002.pdf]

S2 Table. Results of area measurements of one wound shape with Planimator app for different tilt angles with correction switched on or off.

No.	Tilt angle [deg]	Correction (On or Off)	Measured area with Planimator [cm²]
1	0	On	24.797
2	0	On	24.808
3	0	On	24.726
4	0	On	24.763
5	0	On	24.733
6	0	On	24.735
7	0	On	24.814
8	0	On	24.850
9	0	On	24.833
10	0	On	24.791
11	0	On	24.887
12	0	On	24.830
13	0	Off	24.814
14	0	Off	24.759
15	0	Off	24.776
16	0	Off	24.854
17	0	Off	24.887
18	0	Off	24.730
19	0	Off	24.810
20	0	Off	24.842
21	0	Off	24.823
22	0	Off	24.748
23	0	Off	24.805
24	0	Off	24.816
25	5	On	24.804
26	5	On	24.736
27	5	On	24.793
28	5	On	24.728
29	5	On	24.862
30	5	On	24.812
31	5	On	24.738
32	5	On	24.838
33	5	On	24.806
34	5	On	24.733
35	5	On	24.705
36	5	On	24.754
37	5	Off	24.627
38	5	Off	24.587
39	5	Off	24.667

40	5	Off	24.608
41	5	Off	24.623
42	5	Off	24.579
43	5	Off	24.728
44	5	Off	24.644
45	5	Off	24.619
46	5	Off	24.648
47	5	Off	24.604
48	5	Off	24.608
49	10	On	24.823
50	10	On	24.871
51	10	On	24.885
52	10	On	24.883
53	10	On	24.865
54	10	On	24.859
55	10	On	24.759
56	10	On	24.718
57	10	On	24.762
58	10	On	24.798
59	10	On	24.744
60	10	On	24.680
61	10	Off	24.425
62	10	Off	24.480
63	10	Off	24.382
64	10	Off	24.415
65	10	Off	24.479
66	10	Off	24.436
67	10	Off	24.334
68	10	Off	24.348
69	10	Off	24.312
70	10	Off	24.275
71	10	Off	24.237
72	10	Off	24.297
73	15	On	24.816
74	15	On	24.871
75	15	On	24.939
76	15	On	24.841
77	15	On	24.837
78	15	On	24.815
79	15	On	24.678
80	15	On	24.689
81	15	On	24.634
82	15	On	24.768
83	15	On	24.695
84	15	On	24.617
85	15	Off	23.861
86	15	Off	23.908
87	15	Off	23.935

88	15	Off	23.798
89	15	Off	23.849
90	15	Off	23.820
91	15	Off	23.791
92	15	Off	23.869
93	15	Off	23.800
94	15	Off	23.818
95	15	Off	23.727
96	15	Off	23.768
97	20	On	24.555
98	20	On	24.682
99	20	On	24.704
100	20	On	24.765
101	20	On	24.719
102	20	On	24.791
103	20	On	24.759
104	20	On	24.733
105	20	On	24.660
106	20	On	24.745
107	20	On	24.782
108	20	On	24.690
109	20	Off	23.215
110	20	Off	23.125
111	20	Off	23.147
112	20	Off	23.173
113	20	Off	23.244
114	20	Off	23.213
115	20	Off	23.169
116	20	Off	23.102
117	20	Off	23.106
118	20	Off	23.187
119	20	Off	23.198
120	20	Off	23.153
